# Endogenous myoglobin in human breast cancer is a hallmark of luminal cancer phenotype

**DOI:** 10.1038/sj.bjc.6605702

**Published:** 2010-06-08

**Authors:** G Kristiansen, M Rose, C Geisler, F R Fritzsche, J Gerhardt, C Lüke, A-M Ladhoff, R Knüchel, M Dietel, H Moch, Z Varga, J-P Theurillat, T A Gorr, E Dahl

**Affiliations:** 1 Institute of Surgical Pathology, University Hospital Zurich, Schmelzbergstrasse 12, 8091 Zurich, Switzerland; 2 Institute of Pathology, University Hospital of the RWTH, Aachen, Germany; 3 Institute of Veterinary Physiology, Vetsuisse Faculty, University of Zurich, Zurich, Switzerland; 4 Institute of Pathology, Charité – Universitätsmedizin Berlin, Berlin, Germany; 5 Institute of Veterinary Physiology, University of Zurich, Zurich, Switzerland

**Keywords:** breast cancer, myoglobin, oestrogen receptor, fatty acids, luminal type

## Abstract

**Background:**

We aimed to clarify the incidence and the clinicopathological value of non-muscle myoglobin (Mb) in a large cohort of non-invasive and invasive breast cancer cases.

**Methods:**

Matched pairs of breast tissues from 10 patients plus 17 breast cell lines were screened by quantitative PCR for Mb mRNA. In addition, 917 invasive and 155 non-invasive breast cancer cases were analysed by immunohistochemistry for Mb expression and correlated to clinicopathological parameters and basal molecular characteristics including oestrogen receptor-α (ER*α*)/progesteron receptor (PR)/HER2, fatty acid synthase (FASN), hypoxia-inducible factor-1α (HIF-1*α*), HIF-2*α*, glucose transporter 1 (GLUT1) and carbonic anhydrase IX (CAIX). The spatial relationship of Mb and ER*α* or FASN was followed up by double immunofluorescence. Finally, the effects of estradiol treatment and FASN inhibition on Mb expression in breast cancer cells were analysed.

**Results:**

Myoglobin mRNA was found in a subset of breast cancer cell lines; in microdissected tumours Mb transcript was markedly upregulated. In all, 71% of tumours displayed Mb protein expression in significant correlation with a positive hormone receptor status and better prognosis. *In silico* data mining confirmed higher Mb levels in luminal-type breast cancer. Myoglobin was also correlated to FASN, HIF-2*α* and CAIX, but not to HIF-1*α* or GLUT1, suggesting hypoxia to participate in its regulation. Double immunofluorescence showed a cellular co-expression of ER*α* or FASN and Mb. In addition, Mb levels were modulated on estradiol treatment and FASN inhibition in a cell model.

**Conclusion:**

We conclude that in breast cancer, Mb is co-expressed with ER*α* and co-regulated by oestrogen signalling and can be considered a hallmark of luminal breast cancer phenotype. This and its possible new role in fatty acid metabolism may have fundamental implications for our understanding of Mb in solid tumours.

Human myoglobin (Mb) is considered to be one of the best-characterised proteins, with more than 11 200 PubMed-listed publications, since Kendrew and co-workers presented the first three-dimensional model of this molecule in 1958 (Kendrew, 1958). It is commonly described as a cytoplasmic hemoprotein that is solely occurring at milli- to micromolar concentrations in cardiac myocytes and type I and IIa skeletal muscle fibres of mammals. In myocytes, Mb is widely accepted to function as temporary ‘store’ for oxygen (O_2_), able to buffer short phases of exercise-induced increases in O_2_ flux during which it supplies the gas to mitochondria (Ordway and Garry, 2004). Another, more controversially discussed role is Mbs’ facilitation of O_2_ diffusion within muscle cells (Wittenberg, 1970; Jürgens *et al*, 1994). Although Mb knockout mice exhibited normal exercise capacity and no signs of compromised cardiac energetics due to multiple systemic compensations (Garry *et al*, 1998; Gödecke *et al*, 1999), follow-up studies stressed the importance of functional Mb in maintaining nitric oxide (NO) homeostasis in muscle through either scavenging (Flögel *et al*, 2001) or producing the NO molecule (Hendgen-Cotta *et al*, 2008). That way, Mb might participate in tuning vasodilatory responsiveness and protecting the respiratory chain from NO inhibition (Brunori, 2001). Further possible functions of Mb in muscle include synthesis of peroxides (Khan *et al*, 1998), scavenging of reactive O_2_ species (Flögel *et al*, 2004) and binding of fatty acids (FAs) (Masuda *et al*, 2008).

In humans, Mb is synthesised at concentrations of ∼200–300 *μ*M in striated muscle and, although at much lower levels, in a variety of human tumours including medullomyoblastoma (Smith and Davidson, 1984), thymolipoma (Iseki *et al*, 1990), acute leukaemia (Ruck *et al*, 1995) and desmoplastic small round cell tumours (Zhang *et al*, 2003). Following an accidental observation of positive Mb staining in several human carcinomas in 2001, we have systematically examined Mb expression in human breast cancer. Very recently, Flonta *et al* (2009) described Mb in solid tumours including 31 breast cancer cases; however, this small cohort size precluded further statistical analyses. Now, we are presenting the first comprehensive analysis of Mb expression in a large and representative cohort of human breast tissues encompassing normal tissue (*n*=56), ductal carcinoma *in situ* (DCIS, *n*=155), invasive breast cancer (*n*=917) and breast cancer recurrences (*n*=76), enabling a portrayal of the associations between clinicopathological parameters and the range of Mb synthesis seen in mammary carcinomas. We also shed some light on unusual functions of Mb in cancer by looking at the impact of steroids on the steady state level of the protein and by examining Mb's involvement in FA metabolism.

## Materials and methods

### Clinical materials and patients

The matched tumour/normal samples of invasive ductal breast carcinomas and corresponding normal breast epithelium (*n*=10) analysed in this study have recently been described (Veeck *et al*, 2006). For immunohistochemistry, our study included tissue microarrays of normal tissue, intratumoural DCIS, invasive breast cancer and breast cancer recurrences of patients diagnosed at the Institute of Surgical Pathology (University Hospital, Zurich, Switzerland), as described (Theurillat *et al*, 2007). Tumour histology was determined according to the criteria of the (Tavassoli and Devilee, 2003), whereas staging the disease followed (Sobin and Wittekind, 2002). Tumours were graded according to Bloom and Richardson, as modified by Elston and Ellis (1993). Clinicopathological characteristics of the patients/tumours are given in Table 1. For statistical analysis, only cases with clinical follow-up data were considered. The median observation time for overall survival was 59 months for patients still alive at the time of analysis. In total, 225 patients (24%) died during follow-up. Data on adjuvant therapy was not available. Appropriate review board consent has been obtained to allow the use of these materials for research purposes.

### Cell lines

The human mammary epithelial cell line MCF12A, as well as the breast cancerous cell lines BT20, BT474, Cal51, EFM19, HBL100, Hs578T, MDA-MB231, MDA-MB361, MDA-MB453, MDA-MB415, MDA-MB436, MDA-MB468, MCF7, SKBR3, T47-D and ZR75-1 were obtained from the ATCC (Rockville, MD, USA) and cultured under recommended conditions.

### Quantitative real-time reverse transcription PCR

For quantitative PCR experiments, we used the ABI Prism 7500 Fast SDS (Applied Biosystems, Darmstadt, Germany) with the following primers: Mb (111 bp) 5′-GGCATCATGAGGCAGAGATT-3′ and 5′-TCTGCAGAACCTGGATGATG-3′ glyceraldehyde 3-phosphate dehydrogenase (289 bp) 5′-GAAGGTGAAGGTCGGAGTCA-3′ and 5′-TGGACTCCACGACGTACTCA-3′ *β*-actin (185 bp) 5′-GGACGACATGGAGAAAATC-3′ and 5′-ATAGCACAGCCTGGATAGC-3′.

### Western blots

MDA-MB468 cells were harvested with a lysis buffer containing 0.1% NP-40, 400 mM NaCl, 1 mM EDTA (pH 8.0), 10 mM Tris-HCl (pH 8.0) and protease inhibitors. Protein extracts were electrophoresed on a 15% SDS–PAGE gel. Monoclonal antibody mouse anti-Mb (clone Z001, Zymed Laboratories, San Francisco, CA, USA) was used for immunoblotting (1 : 1000 dilution). For equal loading control, blots were stripped and re-probed by monoclonal antibody mouse anti-*β*-actin at a 1 : 5000 dilution (no. A5441, Sigma-Aldrich, Basel, Switzerland).

### Immunohistochemistry and immunofluorescence

Tissue sections were processed using automated immunohistochemistry platforms (BOND, Labvision (Fremont, CA, USA) and Benchmark, Ventana (Tucson, AZ, USA)) using the following antibodies and dilutions: Mb, clone Z001, Zymed Laboratories (1 : 300); fatty acid synthase (FASN), clone 3F2-1F3, Abnova (Taipei, Taiwan) (1 : 2000); HIF-1*α*, clone mgc3, Abcam (Cambridge, UK) (1 : 400); HIF-2*α*, rabbit polyclonal, Novus Biologicals (Littleton, CO, USA) (1 : 150); glucose transporter 1 (GLUT1), rabbit polyclonal, Chemicon (Temecula, CA, USA) (1 : 1000); carbonic anhydrase IX (CAIX), rabbit polyclonal, Abcam (1 : 300). Immunohistochemistry was evaluated by two clinical pathologists (GK, Florian Rudolf Fritzsche). Intensity of Mb, GLUT1, CAIX and FASN was semiquantitatively scored as negative, weakly, moderately or strongly positive (0–3+). Oestrogen receptor-α (ER*α*)/progesterone receptor (PR)/HIFs were evaluated in percent of positive nuclei. Double immunofluorescence (Mb/ER and Mb/FASN) was performed as described (Kristiansen *et al*, 2008).

### Electron microscopy

Tissues from two breast tumours were fixed with 2.5% glutaraldehyde, postfixed with osmium tetroxide, embedded in epoxy resin, cut with an ultramicrotome, mounted on 200 mesh copper grids, stained with uranyl acetate and lead citrate and examined with a Zeiss EM10 transmission electron microscope (Zeiss, Oberkochen, Germany) at 60 kV.

### Expression of Mb gene after incubation of MCF-7 cells with *β*-estradiol

MCF7 cells (8 × 10^5^) were seeded into a 10 cm culture dish, and 24 h later, 17-*β*-estradiol (Sigma-Aldrich, Deisenheim, Germany) dissolved in 5 *μ*l absolute ethanol was applied to the cells at a final concentration of 0 pM, 20 pM, 40 pM and 60 pM. Cells without 17-*β*-estradiol and ethanol served as internal control.

### Expression of Mb gene after incubation of MDA-MB-468 cells with the FASN inhibitor C75

MDA-MB-468 cells (3 × 10^5^) were seeded into six-well plates and exposed to 10 *μ*g ml^−1^ final concentration of the FASN inhibitor C75 (Sigma-Aldrich, St Louis, MO, USA) for 8, 24 and 48 h. The RNA and proteins were isolated according to standard procedures.

### Statistical analysis

Expression data were analysed using the software package SPSS, version 16.0 (SPSS Inc, Chicago, IL, USA). Fisher's exact and *χ*^2^-tests for trends were used to assess the statistical significance of associations between Mb expression and clinicopathological parameters (Table 1). Univariate survival analysis was performed with univariate Cox analyses and Kaplan–Meier curves (Log-rank test). Bivariate correlations according to Spearman were applied to the immunointensity of normal tissue, intraductal and invasive carcinomas.

## Results

### Human breast cancer tissue exerts a complex pattern of Mb expression

#### Mb is detectable on mRNA level in cell lines and human breast tumours

**Figure 1:**
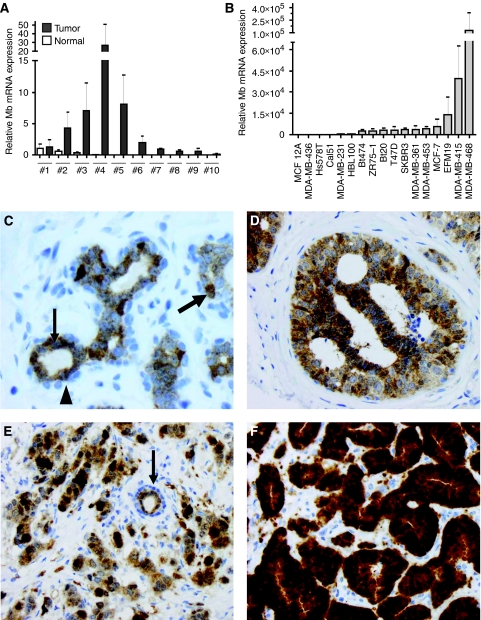
Detection of myoglobin (Mb) mRNA and protein in clinical breast cancer samples and cell lines. (**A**) MB mRNA expression in 10 randomly chosen samples of human breast cancer and corresponding normal breast tissues (no. 1 to no. 10), as measured by quantitative RT-PCR. Calculation of error bars according to Applied Biosystems user manual. Median myoglobin expression was up to 352-fold upregulated in breast tumours (grey) compared with matching normal breast tissues (white). (**B**) Myoglobin mRNA expression in benign MCF12A breast cells and various breast cancer cell lines. (**C**) Normal lobular parenchyma of the breast, illustrating a mild Mb immunoreactivity in luminal epithelial cells (inner layer, thin arrow), whereas myoepithelial cells (outer cell layer, arrowhead) are negative. Note, that single luminal cells (bold arrow) are highlighted by a stronger Mb immunostaining ( × 400). (**D**) Strong myoglobin positivity in a ductal carcinoma *in situ* (DCIS), low grade, tissue showing a slight accentuation of staining in the centre in support of O_2_-diffusion gradients contributing to the Mb expression profile in tissues ( × 200). (**E** and **F**) Examples of invasive ductal carcinoma of the breast (all × 200). (**E**) Moderately differentiated with a moderate-to-strong and patchy Mb expression. In addition, note the central normal duct (arrow) with the Mb-positive secretory cell layer and the Mb-negative myoepithelial layer. (**F**) Strong Mb immunoreactivity in a well-differentiated carcinoma.

Following the initial observation of Mb immunoreactivity in conventional immunohistochemistry analyses, Mb transcript levels were determined in breast biopsies and breast cell lines. Whereas low levels of Mb mRNA were detectable in 4 of 10 cases of healthy breast tissue (Figure 1A), Mb expression was upregulated in 9 of 10 matched normal/tumour tissue samples with a median tumour-to-normal upregulation of 352-fold. With regard to breast cell lines, Mb mRNA was not detectable in benign MCF12A epithelial cells, as well as in MDA-MB436, Hs578T and Cal51 tumour cells (Figure 1B). In all, 10 breast cancer cell lines (MDA-MB231 to MCF7, Figure 1B) expressed detectable but low amounts of Mb mRNA, whereas 3 breast cancer cell lines contained abundant quantities of the Mb transcript, that is, EFM19, MDA-MB415 and MDA-MB468 cells. Regarding the protein abundance, we used a commercial chemiluminescence-based Mb assay and determined the amount of Mb protein present in normoxic 10^6^ MDA-MB468 cells to correspond to ∼65 ng or 4 pmol (which, assuming a mean cell diameter of 20 *μ*m and sphere shaped cells, roughly corresponds to a Mb concentration estimate in the low *μ*M range).

#### Large-scale immunohistochemical analysis of Mb protein in breast tissues

Using a validated monoclonal Mb antibody (Supplementary Figure S1), we next analysed Mb expression on tissue microarrays containing normal tissue (*n*=56), intratumoural DCIS (*n*=155), invasive breast cancer (*n*=917, clinicopathological parameters are given in Table 1) and 76 recurrences of invasive tumours. In normal breast tissue, staining was observed in secretory luminal epithelial cells, but not in myoepithelial cells (Figure 1C, arrowhead), with single secretory cells (bold arrow) staining considerably stronger than adjacent secretory cells (Figure 1C, thin arrow). Altogether, in normal breast tissue, 7% of cases were completely negative for Mb, 49% were weakly positive, 39% stained moderately positive and 5% of tissues stained strongly (Supplementary Figure S2). Ductal carcinoma *in situ* showed altogether a more abundant Mb staining (Figure 1D): 12% of DCIS cases were negative, 38% weakly positive, 39% moderately positive and 11% were strongly positive. In invasive carcinoma, the number of Mb-negative cases was markedly higher than in normal tissue: 29% were negative, 32% were weakly positive, 30% were moderately positive (Figure 1E) and 9% were strongly positive (Figure 1F). A spotted, mosaic-like expression pattern was frequently seen in DCIS and invasive carcinoma (Figure 1E). In tumour recurrences, 30% cases were Mb negative, 22% weakly positive, 33% moderately positive and 15% stained strongly (Supplementary Figure S2). Apparently, although the mean Mb positivity is not significantly altered during malignant progression, an increasing polarisation in Mb expression is noted in the increased number of negative and strongly positive cases from normal tissue to tumour recurrences.

### Electron microscopy: Mb is found without striated muscle elements

As Mb has been described as a marker of rhabdomyoid differentiation, we analysed two breast tumours with strong Mb immunoreactivity by transmission electron microscopy (Supplementary Figure S3A and B). However, no striated muscle elements could be observed in these cases, suggesting that increased expression of Mb occurs independently of rhabdomyoid tumour differentiation.

### Mb expression in invasive breast cancer shows a complex correlative pattern to endogenous markers of Hypoxia (HIF-1*α*, HIF-2*α*, GLUT1 and CAIX)

A subset of invasive breast cancer cases (*n*=150) was additionally analysed for protein level expression of the hypoxia-inducible (transcription) factors 1*α* and 2*α* (HIF-1*α*, HIF-2*α*), which are generally considered as the key regulators of the hypoxic response in cells and tissues, and their down stream targets GLUT1 and CAIX. Myoglobin expression correlated significantly with HIF-2*α* and CAIX, but failed to correlate with HIF-1*α* or GLUT1 (Table 2).

### Tumour-derived Mb expression is linked to oestrogen receptor status and better prognosis, and may represent a marker for luminal-type breast cancer

In invasive breast carcinomas, Mb expression was associated with better histological tumour differentiation according to BRE grading (correlation coefficient (cc)=−0.116; *P*=0.001). Myoglobin did not correlate with pT stage, nodal status or tumour type. Myoglobin expression was positively correlated with ER*α* and PR positivity (cc=0.206, *P*=0.001 and cc=0.180, *P*=0.001) and negatively with the myoepithelial/basal phenotype marker CK5/6 (cc=−0.120, *P*=0.001). No significant correlation was found with Her2, Ki-67 fraction or CD34-microvessel density.

**Figure 2:**
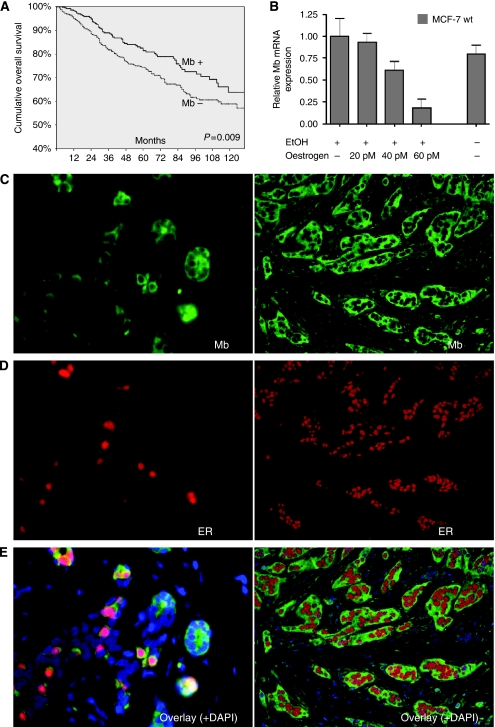
Myoglobin (Mb) expression is linked to better prognosis and oestrogen receptor (ER) positivity and inversely related to oestrogen concentration. (**A**) Kaplan–Meier analysis of a cohort of 917 primary breast cancer cases showing that tumours with high Mb expression (Mb+) show a significant prognostic value for the patient of an improved cumulative overall survival compared with cases with low Mb expression (Mb−). (**B**) Myoglobin expression in MCF7 breast cancer cells is repressed by increasing concentrations of 17-*β*-estradiol (dissolved in ethanol), as measured by quantitative reverse transcription (RT)-PCR. (**C**) Normal breast tissue (left) shows a weak immunofluorescence for Mb in the secretory cell layer, of which some stronger staining cells are intermingled. (**D**) The immunofluorescence for ER*α* (left) shows nuclear staining of single cells also. (**E**)The merged figure (left) illustrates, that these cells, highlighted by ER*α* and Mb, are mostly corresponding (all × 400). (**C**–**E**) This co-expression is also shown in a well-differentiated invasive ductal breast carcinoma (panels on right hand side) (all × 200).

Towards the prognostic value of Mb and clinicopathological parameters, a univariate Cox analysis confirmed histological tumour grade, pT stage, nodal status and hormone receptor (ER*α*/PR) status as significant predictors of overall survival in our patient cohort (Supplementary Table 1). High Mb expression was also significantly associated with longer overall patient survival (5-year survival rate of Mb-positive cases was 83% *vs* 75% in Mb-negative cases, Figure 2A), but lost significance in a multivariate Cox analysis that included pT, pN, BRE-grade, ER*α* and Mb (not shown). In a multivariate survival analysis restricted to ER*α*, PR (as markers of luminal phenotype) and Mb, only PR remained a significant prognosticator (HR=0.62, *P*=0.004), whereas Mb yielded a trend towards a prognostic value (HR=0.75, *P*=0.06) and ER*α* showed none at all (HR=0.74, *P*=0.11). However, in a Kaplan–Meier analysis stratifying tumours in groups that were either (a) negative for ER*α* and Mb, (b) positive for ER*α* or Mb or (c) positive for both ER*α* and Mb, it became apparent that Mb does add some prognostic information to the ER status (Supplementary Figure S4), as tumours that were either positive for Mb or ER showed an intermediate course in comparison with tumours that were negative or positive for both markers (*P*=0.001).

We additionally classified Mb levels in correlation with a publicly available breast cancer expression data set (Farmer *et al*, 2005) (GEO profiles, http://www.ncbi.nlm.nih.gov/sites/entrez, GDS1329). Here, Mb levels are significantly higher (*P*=0.005, Kruskal–Wallis test) in the group of luminal tumours compared with basal or apocrine types. These findings along with the positive correlation of Mb with ER*α*, the better tumour differentiation with improved prognosis and the negative correlation with the basal phenotype marker CK5/6 all point to Mb as a marker of luminal tumour differentiation. This association of Mb with characteristics of the luminal tumour subtype, namely, ER*α* positivity, led us to investigate this point further.

### Mb expression is regulated by oestrogen signalling *in silico* and *in vitro*, and co-localises with the ER

Publicly available DNA array expression data (Geoprofiles, GDS2324) (Coser *et al*, 2003) had already indicated a tight co-regulation of Mb and ER*α* mRNAs in the breast cancer cell line MCF7 by showing that oestrogen starvation was able to induce, while oestrogen application suppressed, Mb and ER*α* transcripts in either a time- or dose-dependent manner (Supplementary Figure S5; ER*α*=ESR1 in figure). We repeated this experiment and could confirm the *in silico* data *in vitro* also, in that application of 17-*β*-estradiol to ER*α*-positive MCF7 breast cancer cells repressed Mb expression dose dependently (Figure 2B).

This co-silencing of ER*α* and Mb expression by oestrogen application to a breast cancer cell line prompted us to analyse the spatial association of both proteins by double immunofluorescence. In normal lobular breast parenchyma, ∼90% of the strongly Mb-positive secretory cells, interspersed in the secretory cell layer that we had already noted in the chromogenic immunohistochemistry, showed a cellular co-localisation (Figure 2E, left) of cytoplasmic Mb (Figure 2C, left) and nuclear ER*α* (Figure 2D, left) staining. In invasive breast carcinomas, this co-expression still was apparent in well-differentiated carcinomas (Figure 2E, right), whereas in moderately–poorly differentiated tumours, this overlap was less prominent (not statistically evaluated).

### Non-respiratory functions of non-muscle Mb?

**Figure 3:**
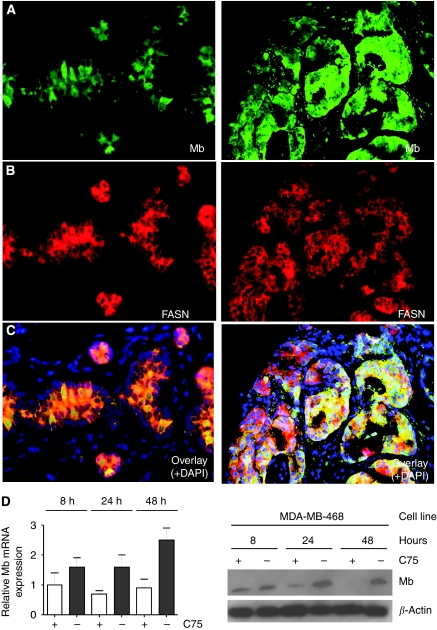
Double immunofluorescence (myoglobin (Mb)/fatty acid synthase (FASN)) staining of breast tissues and regulation of Mb by inhibition of FASN. (**A**) Normal breast tissue (panel on the left) shows weak immunofluorescence for Mb in the secretory cell layer, along with some stronger staining cells that are intermingled. (**B**) The immunofluorescence for FASN (middle) shows strong cytoplasmic staining. (**C**) The overlay figure (bottom) illustrates high degree of correspondence (yellow) between cells expressing Mb and FASN (all × 400). In invasive carcinomas (panel on the right), a greater variation is seen and co-expression of Mb and FASN is only preserved in a subset of cells as the overlay illustrates. In general, the FASN expression exceeds Mb immunoreactivity (all × 200). (**D**) Reverse transcription (RT)-PCR evidence for C75-mediated downregulation of Mb mRNA (left). Western blot evidence for C75-mediated downregulation of Mb protein (right).

The amount of Mb detected in human breast cancer tissue and MDA-MB468 cells was similar in western blot analysis (data not shown) and equalled 65 ng per 10^6^ tumour cells, as described above. As this minute amount of Mb is unlikely to have a significant role in the maintenance of cellular oxygenation, Mb functions alternative to respiratory support have to be considered. Recently, Mb was described as a FA-binding protein and suggested to have a role in the transport of FAs in oxygenated cells (Gloster and Harris, 1977; Yackzan and Wingo, 1982; Masuda *et al*, 2008). This prompted us to investigate the possible co-localisation of Mb and FASN in both healthy and cancerous breast tissue. Fatty acid synthase catalyses the synthesis of unbranched FAs and is upregulated in the broad majority of malignant tumours (Menendez and Lupu, 2007). In a direct comparison of FASN expression with Mb in a subset (*n*=293) of our breast cancer cohort, a highly significant correlation of both proteins was found (cc=0.297, *P*=0.001). Moreover, in normal breast tissues, again, a striking spatial concordance in the expression of Mb and FASN was seen (Figure 3A–C, left), which was also partially retained in cancer (Figure 3A–C, right).

To check for a possible functional association of Mb expression and intracellular FA levels, we used the FASN inhibitor C75. This inhibitor has been characterised as FASN specific, leading to an almost immediate and irreversible enzyme inhibition (Kuhajda *et al*, 2000). In MDA-MB468 cells, we observed a strong time-dependent downregulation of Mb on transcript (Figure 3D, left) and protein level (Figure 3D, right) on FASN inhibition in comparison with control cells, suggesting that non-muscle Mb expression is indeed regulated by intracellular FA levels. Thus, Mb in breast carcinomas and cancer cells might be involved in controlling FA metabolism.

## Discussion

This report is the first systematic examination of Mb expression in a large cohort of breast cancer specimens that allows clinical and molecular correlations and, furthermore, points to unexpected functional facets of this hemoprotein. We first noticed a supposedly aberrant Mb immunoreactivity in a small series of breast carcinomas. To address whether Mb is being actively produced by breast cancer cells or rather taken up from, for example, adjacent musculature, as suggested by Eusebi *et al* more than 25 years ago (Eusebi *et al*, 1984), we analysed its expression both *in vitro* and *in vivo*. On transcript level, we found Mb to be strongly upregulated in breast tumours in comparison with adjacent normal ductal tissue. Myoglobin mRNA was also detectable in breast cancer cell lines, in a small subset, even at surprisingly high levels. This underscored the active expression of endogenous Mb in ordinary invasive ductal breast cancer, which were neither muscle-invasive nor did they, as assessed by light microscopy, exhibit any rhabdomyogenous differentiation. Transmission electron microscopy also did not reveal any striated muscle elements in two strongly Mb-positive breast cancer cases. We therefore conclude that Mb is *de novo* expressed in breast cancer cells, although rare cases of so-called metaplastic carcinomas of the breast might exist in which Mb positivity stems from the rabdomyogenous differentiation of the cells (Jamieson and Rudland, 1990; Yang *et al*, 2003).

On the basis of these encouraging preliminary findings, a large recently described (Theurillat *et al*, 2007) cohort of primary breast cancer, including 917 cases of invasive carcinomas and 155 cases of DCIS, was analysed for Mb expression. Specificity of the Mb antibody was confirmed through the peptide blockage of the immunohistochemical signal (Supplementary Figure S1). In total, 40% of invasive carcinomas showed moderate-to-strong Mb expression and the rate is even slightly higher in DCIS. However, Mb was also consistently seen in mature secretory epithelia of the healthy breast, showing a basal expression in most cells and with a particularly pronounced expression in ER*α*-positive cells, as we noted later. Contrary to earlier reports that found normal epithelia to be invariably Mb negative (Flonta *et al*, 2009), luminal cells in healthy breast clearly have the ability to express Mb at detectable levels. The recently published study of Flonta *et al* (2009), which was published during the preparation of this paper, identified endogenous Mb in various human cancer cell lines and human epithelial tumours including breast cancer and elucidated several signals, including NO, oxidative stress and mitogens (EGF and serum growth factors) and also hypoxia, that were able to stimulate Mb expression in cultivated MCF7 breast cancer cells. However, their study, which encompassed only 31 breast cancer cases, lacked any prognostic or detailed clinicopathological data.

Regarding its occurrence in breast cancer, we found that Mb is preferentially detected in better-differentiated, hormone receptor-positive tumours and is associated with a significantly better prognosis. According to Perou *et al* (2000), all these features are characteristics of the so-called luminal subtype of breast cancer, which shares many molecular similarities with normal secretory epithelia, including a strong expression of cytokeratins typical of mature secretory epithelia (CK8/18), hormone receptor (ER*α*/PR) positivity and negativity for HER2 and the basal cell cytokeratins CK5/6. Further support for considering Mb as a diagnostic marker of the luminal breast cancer subtype comes from the *in silico* analysis of Farmer *et al's* data, in which Mb transcript levels were highest in tumours classified as the luminal subtype (Farmer *et al*, 2005). Our immunofluorescent double stainings further detected co-localisation of cytoplasmic Mb and nuclear ER*α* in secretory cells of normal breast tissue. The same intracellular co-localisation was found in strongly Mb- and ER*α*-positive invasive ductal carcinomas. Beyond this spatial co-existence, Mb and ER*α* transcripts are also co-ordinately and dose-dependently downregulated when MCF7 cells are subjected to an oestrogenic (E2=17-*β*-estradiol) treatment protocol, as shown by *in silico* (Supplementary Figure S5) and *in vitro* (Figure 3B) analyses. In this context, it is of relevance to highlight the observation of Cheng *et al* (2005), who described the inverse relationship of E2 and ER*α* (which we found to be co-expressed with Mb) levels in normal breast epithelia during the mammalian menstrual cycle. These authors further point out that elevated expression of ER*α*, occurring, for example, in postmenopausal women as a result of the loss of E2 signalling, is indicative of non-proliferating breast cells. The hitherto undescribed co-regulation of Mb with ER*α* by oestrogen likely underlies the co-expression of ER*α* with Mb in differentiated luminal cells with their reduced proliferation, which, in turn, might explain the favourable prognosis of Mb-positive tumours. The negative association of tumoural Mb expression and a pre-menopausal status further fits the interpretation of Mb as an oestrogen-silenced gene, as pre-menopausal patients (high oestrogen serum levels) have, in comparison with postmenopausal patients (lower oestrogen serum levels), tumours with lower Mb expression levels. In any instance, as the prognostic value of Mb is limited in comparison with ER or PR, we do not suggest its use as a primary marker to identify luminal phenotype carcinomas for therapy planning. However, Mb might gain clinical relevance if it could be validated as a novel therapy target of cancer, for example, by applying carbon monoxide in sub-toxic concentrations, as has been suggested by Dr Wittenberg at the O2BIP meeting in Aarhus in 2008, which clearly warrants further study. This is more important, as luminal-type breast cancer, although generally more favourable in course, does not respond well to conventional chemotherapy (Bhargava *et al*, 2010).

Myoglobin has different known or alleged functions in muscle tissue including short-term O_2_ storage and buffering, facilitating O_2_ diffusion, scavenging of NO and ROS and also the reverse (peroxidase activity, NO production) and might be involved in FA metabolism (Wittenberg, 1970; Wittenberg *et al*, 1975; Khan *et al*, 1998; Flögel *et al*, 2001, 2004; Ordway and Garry, 2004; Hendgen-Cotta *et al*, 2008; Masuda *et al*, 2008). The functions of Mb in non-muscle tissues are elusive so far. More than 50 years after Thomlinson's and Gray's pioneering discovery to link radiosensitivity with O_2_ tension in tumour tissue (Gray *et al*, 1953; Thomlinson and Gray, 1955), the existence of regional hypoxia in most solid tumours (for review see Vaupel *et al*, 1991; Thews *et al*, 1998; Brown, 1999; Brown and Wilson, 2004) including breast cancer is of high clinical relevance (Vaupel *et al*, 1992). Using the exogenous hypoxia marker pimonidazole, Arcasoy *et al* (2002) concluded from a series of 26 breast cancer cases that 62% of tumours of the breast are pimonidazole-positive and thus hypoxic. As this rate of positivity is in the range of Mb positivity that we observed in breast cancer, we had originally hypothesised to find an association of Mb with tissue hypoxia. In addition, the demonstration of hypoxia-driven Mb induction in a cell line model by Flonta *et al* (2009) fitted this interpretation very well. However, our *in vivo* data, correlating Mb expression with acknowledged endogenous markers of hypoxia, revealed a rather more complex and heterogeneous picture. There was no correlation with HIF-1*α* and its target gene GLUT1, which could either mean that Mb is not significantly regulated by hypoxia *in vivo* or that HIF-1*α* is insufficiently present and active because of the amelioration of tissue hypoxia by expressed Mb. However, given the picomole concentrations of Mb protein that we have measured per million of the maximal Mb-producing MDA-MB468 cell line (Figure 1B), the latter scenario is rather unlikely to explain this lack of correlation. On the other hand, there is a significant correlation of Mb to HIF-2*α* and the HIF-1/HIF-2 target gene *CAIX*, which is in line of at least a partial control of Mb expression by hypoxia. Yet, expression of Mb in breast epithelia can clearly be regulated irrespective of O_2_ levels (that is, signals in normal secretory ductal cells). The fact that Mb in breast malignancies links with HIF-2*α* rather than HIF-1*α* results perhaps from the mutually exclusive status between active HIF-1*α* and ER*α*/PR signalling in breast cancer cells (Kurebayashi, 2003; Massaad-Massade, 2004). In this sense, it is the positive correlation between Mb and ER*α*/PR positivity that might preclude a significant linkage of Mb with HIF-1*α*. All this evidence only underscores the complex regulation of the Mb gene in breast carcinomas, in which hypoxia/HIF signalling is just one of several stimuli to modulate the abundance of this hemoprotein.

To our knowledge, so far only two studies have investigated the functions of Mb *in vivo* models using artificial Mb expression systems. Nitta *et al* (2003) induced Mb expression in hepatocytes by an adenoviral gene transfer in rodents, with the effect that these hepatocytes were significantly more hypoxia resistant. Galluzzo *et al* (2009) were the first to introduce Mb into tumour cell lines and engineered A549 human lung carcinoma cells to ectopically express mouse Mb. Their experimental Mb-expressing tumours displayed reduced or no hypoxia, minimal HIF-1*α* levels, decreased vessel density and finally a more differentiated cancer cell phenotype. In addition, largely suppressed local and distal metastatic spreading was observed. The authors assume that these beneficial outcomes of Mb overexpressing tumours result primarily from the reduction of tumour hypoxia (Galluzzo *et al*, 2009). Although it is tempting to compare their mouse model *in vivo* findings with our *in vivo* observations from human patients, given that higher Mb levels correlate with less aggressive tumour behaviour, both situations are quite different. We have estimated the amount of endogenous Mb in normoxic MDA-MB468 breast cancer cells to equate to ∼65 ng or 4 pmol of Mb protein present in 10^6^ cells. This quantity is certainly far below the high micromolar levels reached by the lentiviral gene transfer (Galluzzo *et al*, 2009). Although such excessive amounts of ectopic Mb in tumour cells can, with reason, be assumed to have a significant impact on tumour respiration and tumour growth, the endogenous picomole quantities of Mb we detected are unlikely to confer meaningful O_2_ storage/buffering capacity to the cell, as already mentioned above. Thus, for these cells, functions of Mb that are not directly linked to the binding and transport of O_2_ have to be considered to understand the physiological relevance of this protein in breast cancer.

One of the functions of Mb that might also be tremendously relevant for tumour cells is the control of FA metabolism. In a multitude of tumours, growth is accompanied with increased FA synthesis, and consequently enzymes catalysing these steps are upregulated and can be used diagnostically and therapeutically (for recent reviews see Menendez and Lupu, 2007 and Mashima *et al*, 2009). We discovered that the co-localisation of Mb with FASN might give a hint towards the FA-binding function of tumour Mb. Fatty acid-binding properties of Mb have been reported and predicted early (Lewis *et al*, 1968; Gloster and Harris, 1977), and have recently gained further attention (Sriram *et al*, 2008). According to Flögel *et al* (2005), lack of Mb in the heart of knockout mice leads to a biochemical shift in cardiac substrate utilisation from FA to glucose oxidation, which, not only corresponds to an adaptive reduction in O_2_ consumption for the equimolar production of ATP but also implicates the protein in providing FA substrates for the mitochondrial *β*-oxidation breakdown *in vivo*. Our indirect demonstration that Mb is likely to be regulated by intracellular FA levels, as shown by the inhibition of FASN, now indicates a putative role for Mb in FA metabolism of cancer cells and clearly warrants further study. Analysing the metabolomic profiles of Mb-positive and Mb-negative breast cancer cases should also yield relevant data to shed more light on the putative role of Mb in FA metabolism.

In summary, Mb is endogenously expressed in normal breast tissue and abundantly in a subset of breast cancer cases. The strong association of Mb expression with presence of the ER*α* explains the generally better prognosis of Mb-positive tumours, compared with tumours lacking Mb. It appears that the regulation of Mb in tumours is so far only incompletely understood. Our data suggests that in breast cancer cells, Mb expression is regulated by oestrogen signalling, possibly also by FA levels and hypoxia. The prospective role of Mb in the lipid metabolism of ER-positive tumours provides a reasonable rationale to investigate this association in further studies, yet the role of other stimuli, for example, NO or growth factors, also needs to be looked at in greater detail. Taken together, these findings further broaden our view on the role of non-muscle Mb that may have fundamental implications for our conception of the biology of solid tumours.

## Figures and Tables

**Table 1:** Clinicopathological parameters of invasive breast cancer cases and relation to myoglobin expression

**Characteristic**	**Number of cases (%)**	**Mb negative**	**Mb weak**	**Mb moderate**	**Mb strong**	***P*-value**
	917 (100%)					
<60 years	416 (45.4%)	125	132	130	29	0.170^a^
⩾60 years	501 (54.6%)	142	157	142	60	
Pre-menopausal	198 (21.5%)	67	64	60	7	0.007^a^
Post-menopausal	719 (78.5%)	200	225	212	82	
Invasive ductal	739 (80.6%)	214	221	228	76	0.375^b^
Invasive lobular	125 (13.6%)	39	49	29	8	
NOS	53 (5.8%)	14	19	15	5	
pT1	335 (36.5%)	91	108	106	30	0.479^a^
pT2	410 (44.7%)	115	134	119	42	
pT3	66 (7.2%)	27	19	17	3	
pT4	106 (11.6%)	34	28	30	14	
pN0	346 (42.5%)	92	107	111	36	0.355^a^
pN1	369 (45.3%)	113	120	105	31	
pN2	69 (8.5%)	21	20	16	12	
pN3	31 (3.8%)	10	8	12	1	
G1	126 (13.7%)	30	40	46	10	0.001^a^
G2	460 (50.2%)	112	155	141	52	
G3	331 (36.1%)	125	94	85	27	
ER negative	163 (18.7%)	78	45	34	6	0.001^a^
ER positive	709 (81.3%)	171	226	231	81	
PR negative	314 (34.8%)	114	104	82	14	0.001^a^
PR positive	588 (65.2%)	145	183	187	73	
HER2—0, 1+, 2+	776 (88.1%)	223	241	239	73	0.346^a^
HER2—3+	105 (11.9%)	33	37	25	10	
Ki-67 ⩽10%	466 (54%)	132	149	139	46	0.985^a^
Ki-67 >10%	393 (46%)	117	115	121	40	
CK5/6 negative	793	212	260	239	82	0.001^a^
CK5/6 positive	96	45	22	26	3	

Abbreviations: ER=oestrogen receptor; Mb=myoglobin; NOS=not otherwise specified; PR=progesteron receptor.

a *χ*^2^-test for trends.

b Pearson's *χ*^2^-test.

**Table 2:** Correlation of myoglobin expression to endogenous markers of hypoxia (cc; *P*-value)

	**HIF-1α**	**HIF-2α**	**GLUT1**	**CAIX**
Mb	0.095; 0.276	0.293; 0.001	−0.039; 0.64	0.286; 0.001
HIF-1α		0.215; 0.020	0.334; 0.001	0.285; 0.001
HIF-2α			−0.206; 0.024	0.223; 0.015
GLUT1				0.252; 0.003

Abbreviations: CIAX=carbonic anhydrase IX; GLUT1=glucose transporter 1; HIF=hypoxia-inducible factor.
