# Supplementary material for: Endogenous myoglobin in human breast cancer is a hallmark of luminal cancer phenotype
Source: Br J Cancer. 2010 Jun 8;102(12):1736–45. doi: 10.1038/sj.bjc.6605702 (PMC2883703; doi:10.1038/sj.bjc.6605702)
Supplement: Supplementary Figures [file 6605702x1.ppt]

## Slide 1
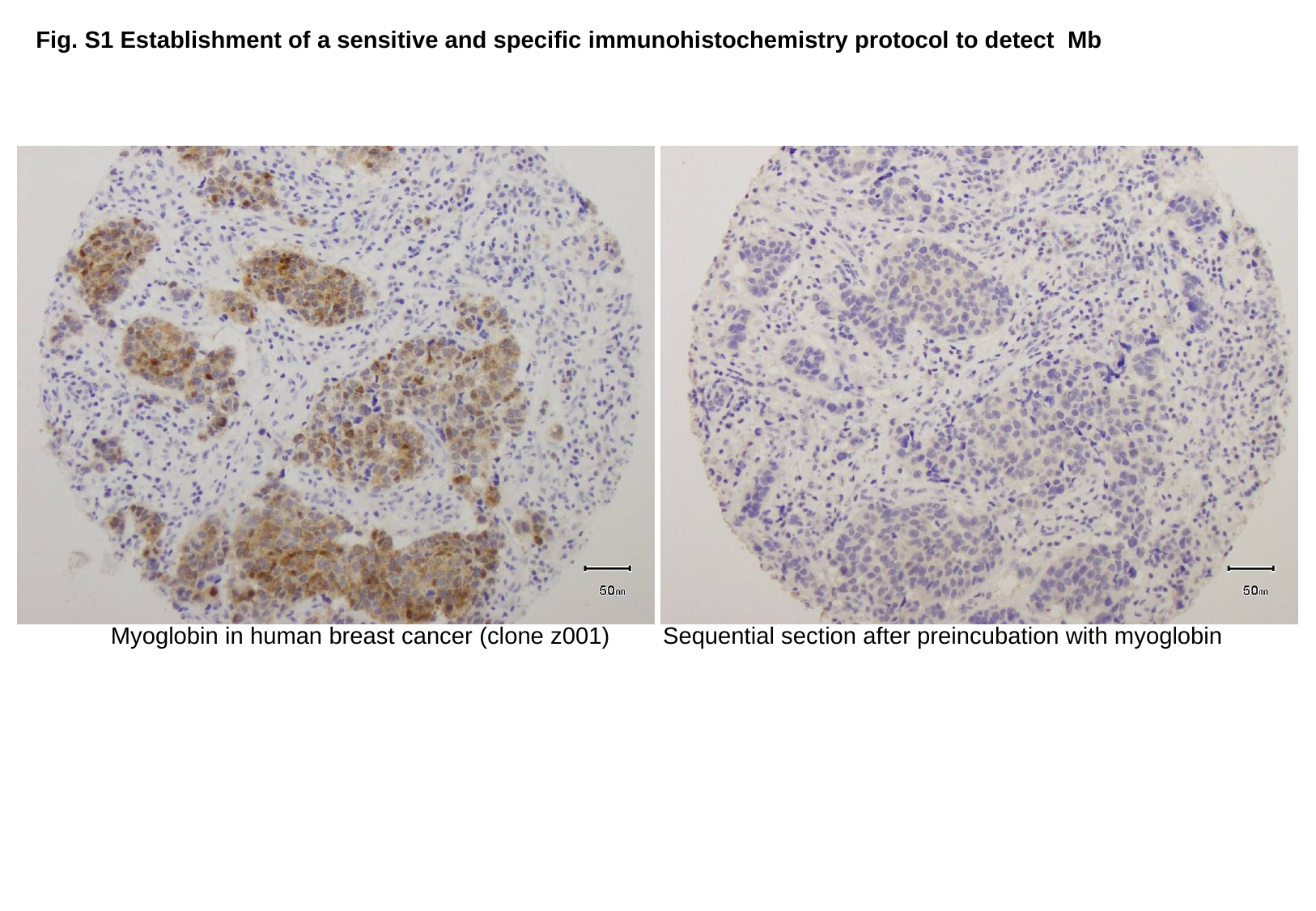

Fig. S1 Establishment of a sensitive and specific immunohistochemistry protocol to detect Mb
Myoglobin in human breast cancer (clone z001) Sequential section after preincubation with myoglobin

## Slide 2
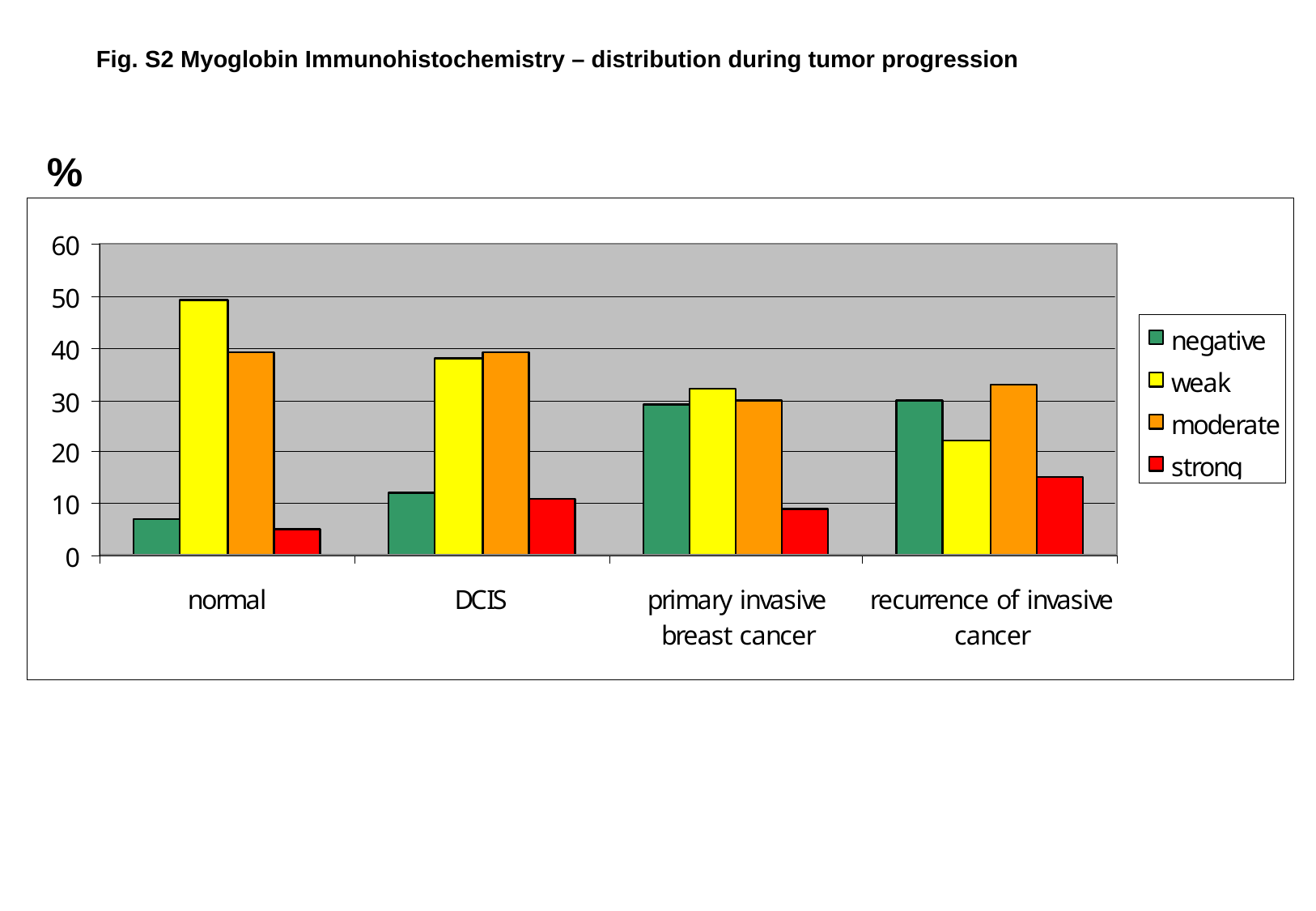

Fig. S2 Myoglobin Immunohistochemistry – distribution during tumor progression
%

## Slide 3
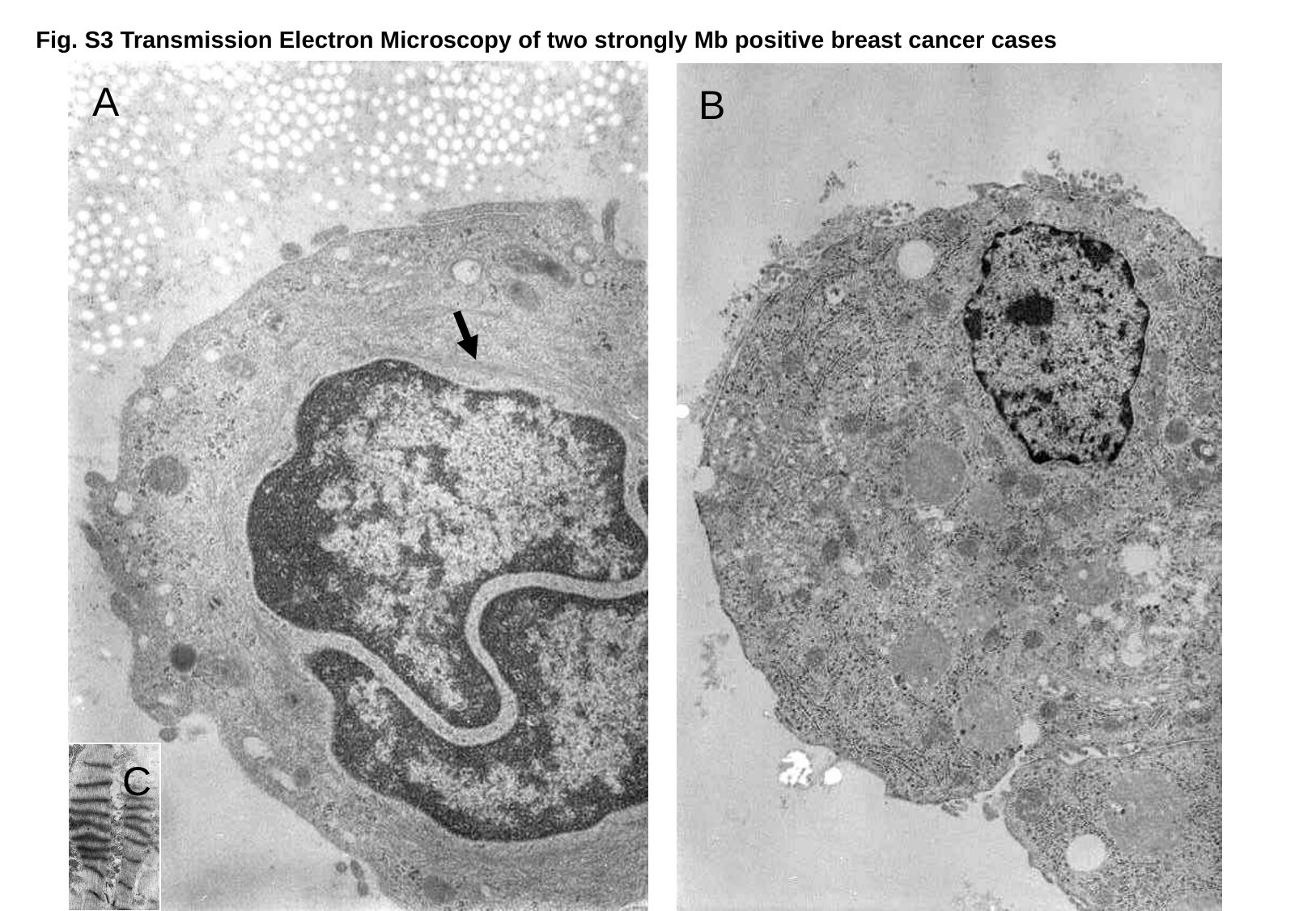

Fig. S3 Transmission Electron Microscopy of two strongly Mb positive breast cancer cases
A
B
C

## Slide 4
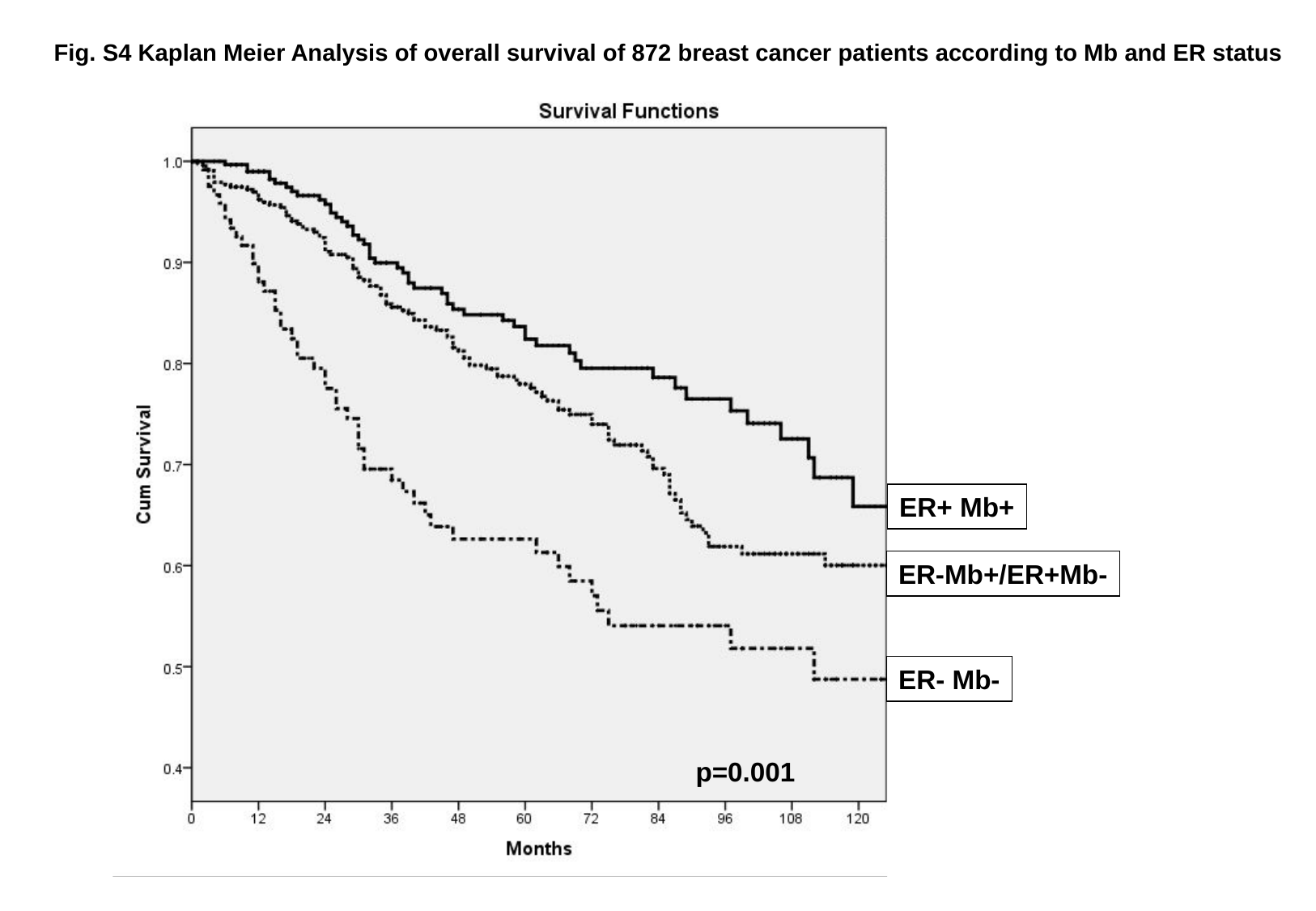

Fig. S4 Kaplan Meier Analysis of overall survival of 872 breast cancer patients according to Mb and ER status
ER+ Mb+
ER-Mb+/ER+Mb-
ER- Mb-
p=0.001

## Slide 5
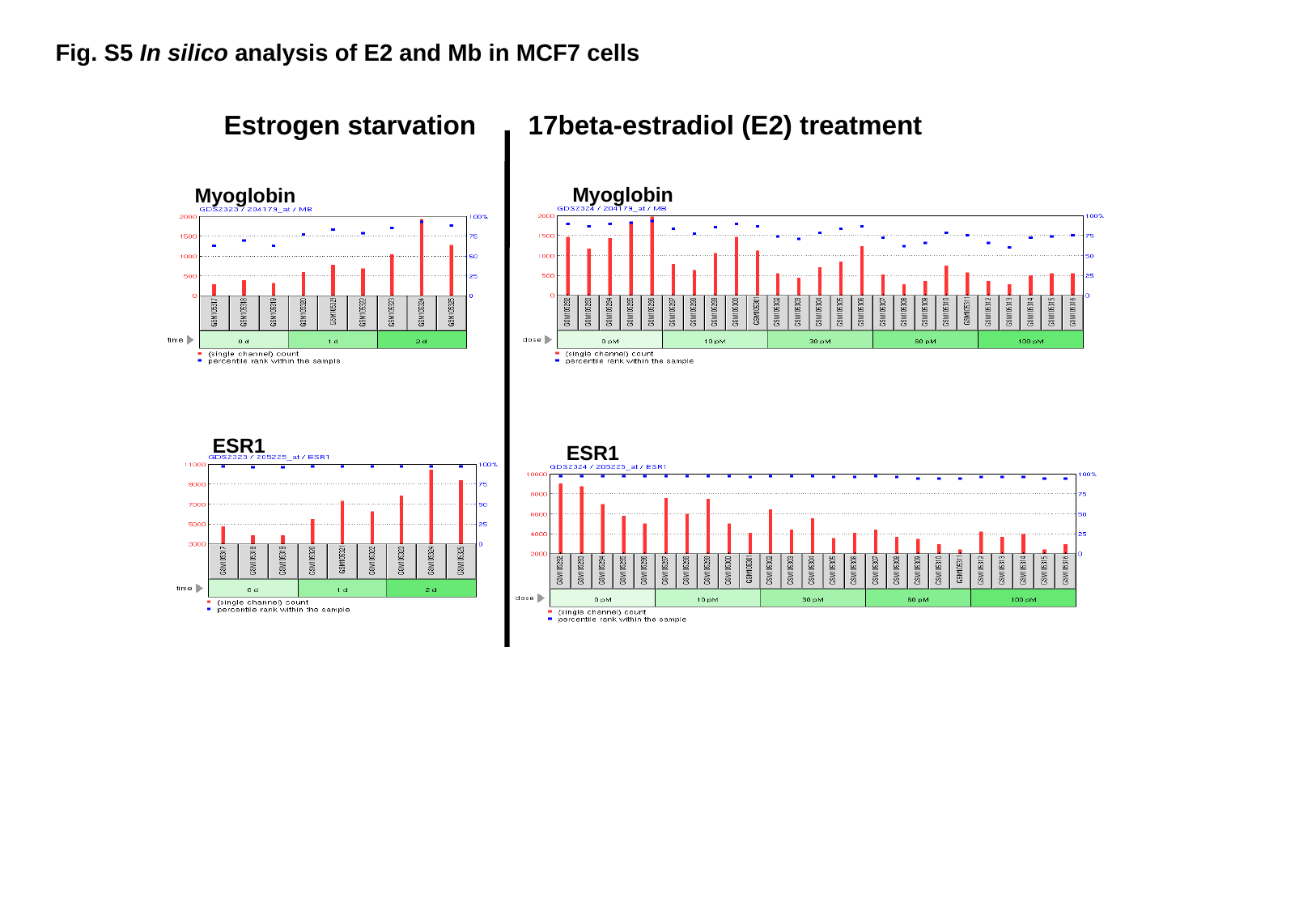

Fig. S5 In silico analysis of E2 and Mb in MCF7 cells
Estrogen starvation 17beta-estradiol (E2) treatment
Myoglobin
Myoglobin
ESR1
ESR1
