# Supplementary material for: Endogenous myoglobin in human breast cancer is a hallmark of luminal cancer phenotype
Source: Br J Cancer. 2010 Jun 8;102(12):1736–45. doi: 10.1038/sj.bjc.6605702 (PMC2883703; doi:10.1038/sj.bjc.6605702)
Supplement: Supplementary Figure Legends [file 6605702x2.doc]

*Supplemental Legends.*

*Fig. S1. Establishment of a sensitive and specific immunohistochemistry protocol to detect myoglobin*

Three different myoglobin antibodies were compared for their suitability to detect myoglobin in formalin fixed paraffin embedded (FFPE) tissues, i.e. mouse monoclonal clone z001, Zymed, USA; mouse monoclonal clone MG1, Neomarkers, USA and rabbit polyclonal anti-human myoglobin, DAKO, Denmark. All three antibodies yielded comparable results with clone z001 showing the cleanest immunoreactivity. To further enhance sensitivity a heat induced epitope retrieval (HIER) step was included in the z001 detection protocol (left). A blocking experiment with purified human myoglobin (1:10 mol) confirmed the specificity of the immunoreaction (right).

*Fig. S2. Mb immunohistochemistry – distribution of expression during tumor progression*

The distribution of intensity of Mb immunoreactivity, categorized as negative (green), weakly (yellow), moderately (orange) and strongly positive (red) in normal breast tissues, DCIS, invasive breast cancer and recurrences of invasive breast cancer is shown. Clearly, on average no simple down or upregulation ob Mb expression is noted, but a polarization with increasing losses and gains in intensity of Mb expression with tumor progression is apparent.

*Fig. S3. Transmission electron microscopy of two Mb positive breast cancer specimens*

Since myoglobin has been described as a marker of rhabdomyoid differentiation, two breast tumors with strong myoglobin immunoreactivity were analysed by transmission electron microscopy. **A)** Cell of an invasive lobular carcinoma with broad bundles of intermediate filaments (arrow). **B)** Cell of an invasive ductal carcinoma exhibiting a plethora of endoplasmatic reticulum. No striated muscle elements as exemplified in **C)** (human heart muscle) were noted.

*Fig. S4. Kaplan Meier Analysis of overall survival of 872 breast cancer patients with respect to Mb and ERα status*

This figure illustrates the additive prognostic value of Mb and ER by comparing tumors negative for ER and MB (half-dotted line), tumors positive for ER and Mb (full line) and tumors which are positive for either, ER or Mb (dotted line). Note, that the latter group that has positivity for one marker indicative for luminal phenotype does do considerably better than tumors negative for both, however, it does not quite reach the relatively favourable course of the group in which both markers are positive.

*Fig. S5. In silico analysis of the influence of estrogen signaling on Mb and ESR1 in MCF-7 cells*

Retrieving the array datasets of Coser *et al.* (2003) via the web based data mining tool GEO Profiles, two experiments were analysed. On the left, the influence of estrogen starvation on the expression of Mb and ESR1 (=ERα) is illustrated. Clearly, a concomitant up-regulation of both transcripts (single channel counts, red bars) during 48h is apparent. Conversely, the profiles of MCF-7 cells that were treated with 17-β-estradiol (on the right) show a dose dependent down-regulation of Mb and ESR1. Note, however, that the amplitude of either ESR1 regulation (red bars) is artificially enhanced due to the start of Y-axis labels at non-zero values (explains poor correspondence with blue percentile rank data in ESR1 profiles).
