# Supplementary material for: Endogenous myoglobin in human breast cancer is a hallmark of luminal cancer phenotype
Source: Br J Cancer. 2010 Jun 8;102(12):1736–45. doi: 10.1038/sj.bjc.6605702 (PMC2883703; doi:10.1038/sj.bjc.6605702)
Supplement: Supplementary Table 1 [file 6605702x3.doc]

Supplemental Table 1. Univariate Cox survival analysis of clinico-pathological parameters and myoglobin expression

| Parameter | Relative risk | 95%-Confidence interval | p-value |
| --- | --- | --- | --- |
| Age (<= 60 *vs.* >) | 1.881 | 1.495-2.367 | 0.001 |
| Menopausal status | 1.569 | 1.172-2.100 | 0.002 |
| pT (1-4) | 1.699 | 1.537-1.877 | 0.001 |
| pN (0-3) | 1.610 | 1.403-1.846 | 0.001 |
| G (1-3) | 1.616 | 1.360-1.920 | 0.001 |
| ER (neg. *vs.* pos.) | 0.549 | 0.425-0.709 | 0.001 |
| PR (neg. vs. pos.) | 0.527 | 0.408-0.680 | 0.001 |
| HER2 (0-2+ *vs.* 3+) | 1.839 | 1.372-2.465 | 0.001 |
| CK5/6 (neg *vs.* pos) | 1.669 | 1.166-2.390 | 0.005 |
| MB^*^ | 0.686 | 0.516-0.913 | 0.010 |

^*^ dichotomized by the median
